# Xylosyltransferase 2, a protein encoded on chromosome 17q, is involved in HTLV entry

**DOI:** 10.1186/1742-4690-8-S1-A201

**Published:** 2011-06-06

**Authors:** Stig M R Jensen, Kathryn S Jones, Laurence Bénit, Daniel C Bertolette, Cari Petrow-Sadowski, Francis W Ruscetti, Claudine Pique

**Affiliations:** 1 Cancer and Inflammation Program, NCI-Frederick, Frederick, Maryland, USA; 2 Basic Research Program, SAIC-Frederick Inc., National Cancer Institute-Frederick, Frederick, Maryland, USA; 3 Institut Cochin, Paris, France; 4 Université Paris Descartes, Paris, France

## 

Early studies mapped the entry receptor for HTLV-1 and HTLV-2 to the long arm of chromosome 17. However, later observations that the titer of HTLV Env-pseudotyped viruses was low on some mouse-human hybrid cell lines containing human chromosome 17q led to the belief that this gene was not involved in HTLV infection. Recent studies showing that efficient HTLV-I infection requires three molecules (GLUT-1, NRP-1, and HSPGs), prompted us to examine whether a gene on chromosome 17 is related to this receptor complex.

Since NRP-1 or GLUT-1 map to other chromosomes, we examined chromosome 17q for genes involved in the synthesis of HSPGs. A gene at 17q21.33, XYLT2, encodes Xylosyltransferase 2 (XT-II), one of two isoforms of an enzyme involved in glycosaminoglycan-chain modifications on proteoglycans. Studies revealed that both the titer of HTLV-1 pseudotyped virus and the level of binding of soluble HTLV-1 SU proteins were dramatically lower on a cell line lacking functional xylosyltransferase (CHOK1-745) than on the parental line (CHOK1). Expression of transgenic XT-II in CHOK1-745 cells increased the levels of HTLV-1 SU binding and the titer of HTLV-1 Env pseudotypes to levels similar to those in wild-type cells. The role of XT-II in HTLV-2 and HTLV-3 Env-mediated entry is being investigated, as well as the role of xylosyltransferases in the infection of T and dendritic cells by HTLVs. These data provide molecular evidence for the previously reported association of a chromosome 17-encoded product in HTLV-1 entry and confirm the critical role of proteoglycans in this process.

